# Reframing the sex debate in scientific research: three frameworks to support rigor without rigidity

**DOI:** 10.1186/s13293-025-00808-2

**Published:** 2025-12-19

**Authors:** Stacey A. Ritz, L. Zachary DuBois

**Affiliations:** 1 Department of Pathology & Molecular Medicine, Faculty of Health Sciences, McMaster University, Hamilton, Canada; 2 Department of Anthropology, University of Oregon, 1218 University of Oregon, Eugene, OR 97403 USA

**Keywords:** Sex, Binary sex, Dynamic system, Gender, Continuum, Operationalization, Spectrum

## Abstract

Debates about the nature of sex are prominent in scientific, scholarly and public discourses. Scientific as well as societal debates largely focus on whether sex is appropriately conceptualized as a binary, a spectrum, or some other form. These have taken on particular urgency because of highly contested, polarizing, and consequential developments in the global landscape in which the political weaponization of binary sex has escalated. Here we propose three frameworks useful for retaining focus on *why* sex might be conceptualized in a certain way to address the aims of a given project to help avoid pitting binary versus non-binary approaches against one another in a continued impasse. These three frameworks are (a) sex as a system of classification, (b) sex as a trait, and (c) sex as a dynamic system. We also provide a series of questions and considerations to guide a critical read of scholarship and scientific engagement of sex in ways that might help mitigate the isolating effects of disciplinary silos and advance cross-disciplinary dialogue and collaboration. Thus, rather than recapitulating discussions of how sex should be conceptualized or trying to establish a universally applicable definition of sex, these three frameworks and guiding questions can be drawn on to consider *why* certain projects might employ particular definitions and methods of operationalization of sex over others, and outline the merits and drawbacks of each. By refocusing our scholarly attention on what sex is empirically being employed to do and why, and providing these frameworks to think with, we hope to bring some essential bridging between those for and those against binary thinking about sex and foster a diversity of approaches that might more effectively build on one another rather than exist in opposition. We can then continue to try to nimbly engage what it is sex is being leveraged to do and why in order to ask how a given conceptualization will help us to learn about a given phenomenon.

## Highlights

Scientific and societal debates largely focus on whether sex is appropriately conceptualized as a binary, a spectrum, or other form. This is a pressing issue as political weaponization of binary sex continues to escalate.

We offer a series of questions and three frameworks to guide a critical read of scholarship and scientific engagement of sex: (a) sex as a system of classification, (b) sex as a trait, and (c) sex as a dynamic system.

Drawing on these frameworks could help to facilitate nimble engagement with *what* sex is being leveraged to do and *why*, reorient us to ask *how* a certain conceptualization could inform a given phenomenon, and thereby avoid pitting binary versus non-binary approaches against one another in a continued impasse.

## Introduction

Ongoing debates among scientists, scholars, and society at large, about the nature of sex largely focus on whether sex is best conceptualized and operationalized in binary terms or as something more fluid, as for example, a spectrum or mosaic. These debates are clearly relevant in the context of historical and ongoing politicization of sex and the myriad ways it has been weaponized to discriminate and oppress. But too often, there seems to be a tendency to equate the methodological operationalization and variation in different disciplines and research contexts as though they (individual scientists, teams, or even ‘schools’ of thought) are staking a universal claim for what sex *is* and how it should *always* be operationalized. From our standpoint, this assumption is part of the problem. It is clear that there is broad recognition of the complexity of sex and the role of human decision-making in how biological variation often becomes categorically operationalized. The title of this special issue, *Eschewing the Binary*, invites us to scrutinize the very processes underlying how the concepts of *sex* and *gender* themselves *become* binary, and also gestures to the proliferation of scholarship that specifically problematizes the female-male binary that is so predominant in structuring how we think about sex and gender. Here, rather than interrogating whether sex is (or is not) a male-female binary, we consider why the debate itself seems so intractable and suggest a reorientation to promote new ways forward.

Sexual reproduction is clearly highly relevant for understanding so much about biology, human variation, and lived experience, and there are some aspects that are well-explained or characterized in binary terms. At the same time, pervasive imposition of a binary conceptualization of sex can and does do great harm when socioculturally and politically enforced including through stigma, transphobia, rigid gender norms, discriminatory legislation, and other forms of structural sexism. As scientists, we have both found it challenging to live in this tension. Here, we briefly review varied perspectives and offer three frameworks to help guide dialogue about sex conceptualization and its usage moving forward. It is our hope that new opportunities could emerge to unpack the varied reasons why researchers draw on different frameworks, whether binary, spectrum, mosaic, or otherwise. This could help make explicit some often-implicit tensions impeding cross-disciplinary dialogues.

Our perspective is inspired by our recent experience at a forum specifically designed to engage the idea of gender and sex *entanglements*, which gathered over 30 participants from a range of biological, health, and social science disciplines [1]. The forum aimed to bridge disciplinary silos and entrenchments and provide space for novel discussion of sex, gender, and gender/sex entanglements to advance scientific practice, culminating in an open access edited volume reflecting those discussions [1, 2]. As scholars focused on the application of sex and gender considerations in health research, in our own writings we have each challenged rigid, binary conceptualizations of sex [3–5]. We find strong alignment with others pushing forward entangled and contextualized approaches, and who have pointed to the limitations of trying to pursue or impose universally applicable definitions or means of operationalization for sex and gender (e.g. [6–12]). At the same time, in this paper we are focusing exclusively and deliberately on the concept of sex alone, because there continues to be a prevailing discussion that centers around the nature of sex - what it *is* - whether binary or otherwise. We think this continued focus reflects a need to advance several overlapping conversations moving forward including how sex is constituted in scientific discourse but also more directly engaging what sex does *politically* in specific state-level projects, echoing Currah [13, 14] in the context of human gender/sex diversity.

Toward this end, we offer here a series of questions and three frameworks to guide a critical read of scholarship and scientific engagement of sex in ways that might help mitigate the isolating effects of disciplinary silos and advance cross-disciplinary dialogue and collaboration. Application of these may facilitate nimble engagement with *what* sex is being leveraged to do and *why*, and reorient us to ask *how* a certain conceptualization could inform a given phenomenon, and thereby avoid pitting binary versus non-binary approaches against one another in a continued impasse.

## The political misuse and abuse of science and “sex”

The contemporary political landscape is one where certain binary definitions of sex are actively being weaponized for political gain and scientific authority is used as justification for discrimination. These draw on and codify extremely narrow, scientifically inaccurate definitions of sex, as in the claims about gametic sex *at conception* made in the US President’s Executive Order (EO) from 20 January 2025. Titled, *Defending Women from Gender Ideology Extremism and Restoring Biological Truth to the Federal Government* [15], the EO attempts to wield the authority of science through the repeated use of terms like “biological truth,” “biological reality,” “biological facts,” “biological classification,” the “biological category of sex,” and “biologically distinct sexes” in order to imply that these claims are ‘objective’ and ‘true’ (as opposed to the ‘ideological extremism’ it imputes to other conceptualizations of sex and gender). This EO is part of a larger set of actions to undermine scientific and biomedical research pertaining to sex and gender and research inclusive of sexual and gender minority people, with the termination of already-approved and -funded grants pertaining to sex and gender [16–20].^1^ Around the same time, in April 2025, the Supreme Court of the United Kingdom issued a ruling that the legal definition of a woman is based on “the sex of a person at birth” (i.e. assigned female at birth) [22]. As these examples illustrate, discussions about sex and gender are anything but abstract, and methods of operationalization in research, policy, and practice have significant real-world effects.

These larger overarching, societal-level debates about sex, clearly have implications for research practice, and although the basis for a male-female binary is contested, there is no doubt that sex and gender categories structure many prominent aspects of human social life and organization. The apparent intractability of these issues has the unfortunate effect of entrenching silos between disciplines, moralizing divides, and undermining the capacity for types of collaborations that are arguably key for advancing science and scholarship in these areas. Even prior to the EO and rulings in the UK and elsewhere, the politicization and misuse of “sex” have been exposed by many scientists and scholars in scientific commentaries and the popular press (e.g. [23–26]) and have inspired several to push for ways to center gender and sex more effectively in scientific research with attention to implications for policy (e.g. [27, 28]).

Importantly, many scholars in the biological sciences are politically progressive on social questions of gender inclusivity, and several who examine binary sex differences have been explicit that they are motivated by a desire to combat the exclusion of women in research. Moreover, some scientists have explicitly opposed the way that binary sex is used to support discriminatory policies or practice (e.g. [29, 30]). At the same time, the contention that ‘sex is not binary’ can be harder to reconcile with biologies of sexual reproduction such as anisogamy (the existence of two discrete forms of gametes) and forms of dimorphism related to reproduction (like the presence or absence of a uterus, testes, or ovaries). For many, viewing sex as a male-female binary aligns comfortably with a certain cultural commonsense that structures enormous parts of our social lives around these categories, and with certain biological traits that are closely associated with these categorizations. However, it is also very clear that a female-male binary framework is not only inadequate to (fully) explain sex-related variation but is also used to justify systems of oppression and inequity. These tensions have implications in the general social discourse and for scientific research.

The present-day calls from various institutions to incorporate sex and gender considerations in science arise from recognition that their historic omission in research created significant gaps in knowledge with wide-ranging consequences [31–34]. Since the early 1990 s, several mandates aiming to combat the lack of attention to sex and gender and the relative underrepresentation of women and female animals from research in many fields have required researchers to address sex and gender-related considerations in their work [32, 35–46], motivating a substantial amount of scientific focus on binary male-female sex comparisons. At the same time, (and building on decades of feminist interventions in science about sex), scholarly discussion about how sex is conceptualized and should be operationalized has taken on new life. This includes for example contemplating the entanglement of sex and gender, aiming to be more inclusive of gender/sex diversity [47], suggesting alternative frames to the cis/trans binary of gender [48], and raising valuable questions challenging us to carefully consider the very relevance of sex [49, 50].

Present debates are often centered on the question of whether a “male versus female” binary is an appropriate or useful conceptualization, as vividly reflected in articles with titles like *“Challenging the binary”* [3], *“Transcending the male-female binary in biomedical research”* [4], *“Biology is not binary”* [24], “*Male-female comparisons are powerful in biomedical research – don’t abandon them”* [29], *“Biological sex is binary*,* even though there is a rainbow of sex roles”* [30], *“Is ‘sex’ a useful category?”* [49], *“Sex eliminativism”* [50], “*Is sex still binary?”* [51], *“Breaking binaries leads to a better understanding of ecology and evolution”* [52], *“Deconstructing sex: Strategies for undoing binary thinking”* [53], *“Biology should not dispense with sexes”* [54], *“No way out of the binary”* [55], and *“No bones about it: Sex is binary”* [56]. While their evocative titles give the impression that they are at odds with one another, there is a lot of common ground: those that ultimately end up arguing for a male-female binary conceptualization generally recognize that there is complexity and variation in sex, and those that end up arguing against a binary generally recognize that at least some of the characteristics associated with sex are dimorphic. Regardless of where they land on the question at the end, the authors of these papers all aim to reconcile the tension of the complexities of sex in ways that make sense for their scholarly purposes.

Understood in that way, we can see value in both positions: it can be simultaneously true that using a male-female comparisons can be powerful in biomedical research for certain purposes and also true that challenging the binary and using alternative framings can lead to more sophisticated understanding of biological phenomena. In fact, we would argue that the biomedical researcher who makes use of male-female comparisons in their experimental design has scope to challenge the binary in the ways they analyze and interpret those binary categorical comparisons. For example, when conducting male-female comparisons, we have an opportunity to be deliberate about drawing attention to heterogeneity, overlap, variation, and mechanism, and to invoke non-cis-normative conceptualizations and explanations for our findings. In other words, these do not need to be mutually exclusive ways of engaging questions about sex and gender. Nor do we need to pit biological and evolutionary ways of thinking about sex and gender against explicitly feminist and critical approaches; they can co-exist and mutually inform one another in fruitful ways [47, 57, 58].

The three frameworks: Sex as a system of classification, a trait, and a dynamic system.

**Fig. 1:**
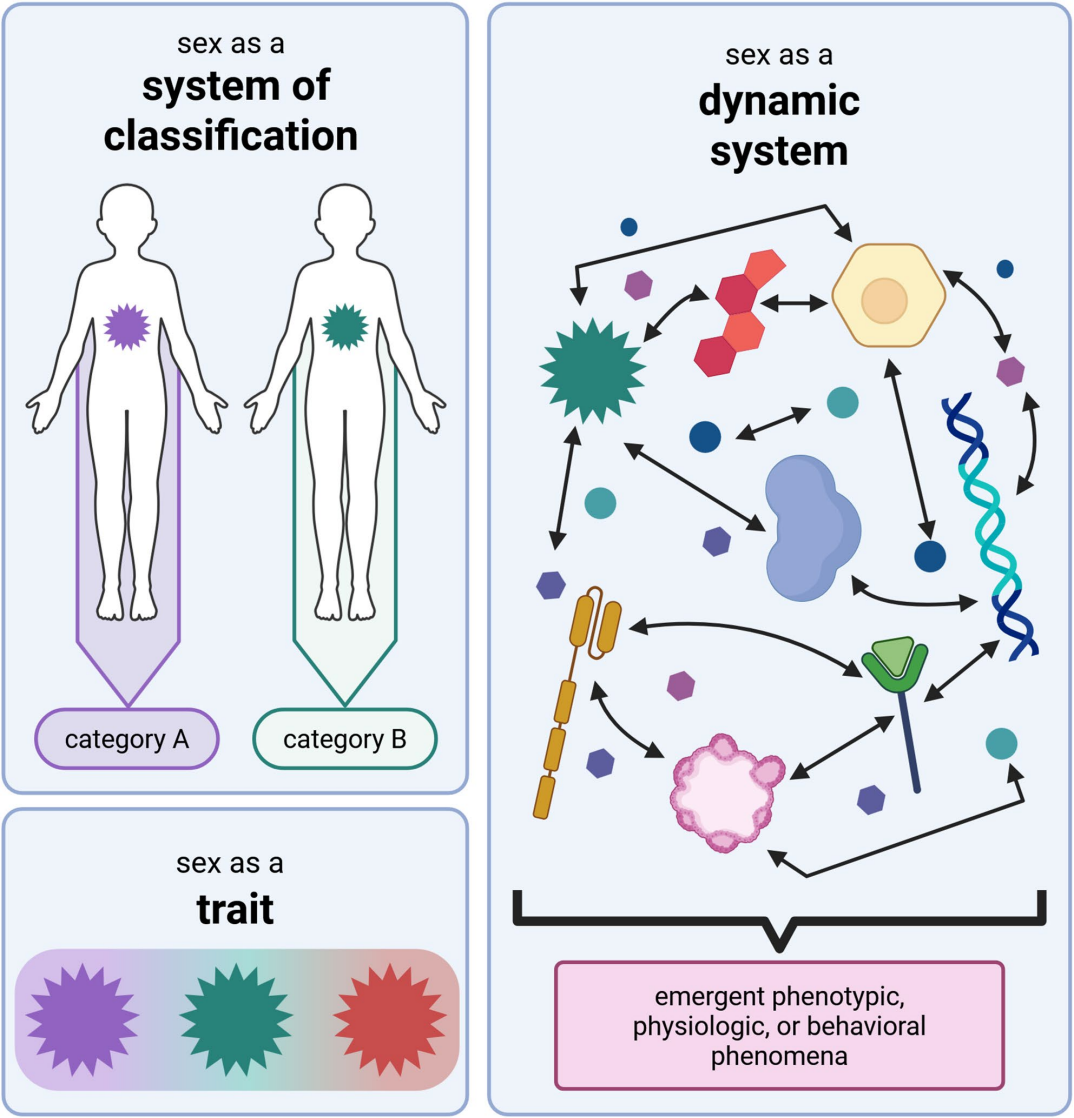
-Sex as a system of classification, a trait, and a dynamic system. Sex can be understood as a system of classification - that is, a structure of logics that are used to assort individuals into different categories based on an attribute or set of attributes, with the categories being the locus of operationalization, comparison, and analysis; the most common such categorization is a male-female binary, but other categories are also possible. A second framework is to operationalize sex with respect to a trait, where a certain attribute is the locus of operationalization, comparison, and analysis. Such a trait can be discrete (lending itself to a binary or other-numbered structure) or continuous (more like a spectrum). A third mode understands sex as a dynamic system, a collection of multiple elements that interact with one another and respond to stimuli to produce an emergent phenomenon that we regard as *sex;* these elements can include all manner of molecular, cellular, genetic, hormonal, or neuronal inputs, which also network to stimuli from the external environment as well. This mode emphasizes attention to the mechanisms that underlie the phenomena we associate with sex and which explain sex-related variation, and the complexity of their entanglement and interaction. Figure created using BioRender

In reflecting on the various implicit and explicit discourses in the literature, here we provide three frameworks for reflecting on how and why sex might be operationalized as (a) a system of classification, (b) a trait, or (c) a dynamic system (Fig. 1). In other words, instead of having a conversation about whether or not sex is itself binary, these frameworks help guide a shift to discussion of why sex is being invoked in a particular context (whether scientific or in day-to-day life) and downstream implications of any given approach. Our hope is that these frameworks could help us develop and describe our own work with greater transparency and more clearly understand and evaluate the work of others. At the same time, we appreciate the statistician George Box’s insight that “all models are wrong, but some are useful” [59], and indeed each of these frameworks for understanding sex are both useful and wrong in their own ways.

### Sex as a system of categorical classification

This is the most common framework, one employed by medical professionals in assigning sex at birth, by researchers creating groups for experimentation or analysis, and arguably is also how individuals subconsciously categorize one-another in day-to-day life based on culturally recognized attributes. When sex as a system of classification is guiding a given perspective or project (whether consciously or not), sex is operating as a structure of logics that are used to assort individuals into those categories.

Generally, categories are developed and used to help us make sense of complexity by imposing a structure for organizing aspects of reality and experience. To effectively do this, the operationalization of sex as a system of classification involves at least three different decisions: (a) selecting the characteristics deemed pertinent for categorization; (b) establishing the number of categories (and usually their names); and (c) demarcating the boundaries used to define membership in each category. It is crucial to highlight that -- unlike characteristics themselves -- categories are not themselves facts or things that are *discovered* or *identified*, but are conceptual entities that are *created* via this decision-making process. Using sex in a categorical sense reflects particular perspectives about what matters in a given context, since there are alternative ways of dividing and naming categories, and alternative characteristics that could have been drawn on to draw bounds around those categories. Because of this, the basis for sex classification may differ from one context to another by utilizing different categories, characteristics, or demarcation points.

Gametes are a useful example here. We can say that it is a *fact* that there are individuals whose bodies produce ova and individuals whose bodies produce sperm, but the decision to create 2 categories, call them ‘male’ and ‘female’, and base the categorization of individuals on the single characteristic of gamete type is a set of *decisions.* In some cases, it may indeed make sense to use the male-female categories with criteria for categorization based on gamete type. In other cases, it may be more relevant to use the same male-female categories but use a different characteristic - such as genital morphology - to make the categorization; unlike with gametes, where only two forms exist, genital morphology has considerably more variation, and so using this criterion requires more careful consideration about where to draw the distinction between categories. Continuous variables could be used as the basis for group-level categorization by defining a threshold value to define membership in one category or another, for example, estrogen levels could be used to define high, medium, and low estrogen categories. Other contexts might conceivably call on sex as a system of classification based on a combination of several characteristics rather than just one, or rely on a different number of categories named differently.

Categories (whether binary or otherwise) are particularly useful when the goal is to make a comparison and delineate differences between them. This is in part because categories themselves are intrinsically defined based on difference, and so the creation of categories presupposes difference between them. Research questions based principally on a male-female binary categorization are probably most useful when the relevance of sex and gender are underrecognized and understudied. In contrast, when male-female comparisons are already well-documented, drawing on the *trait* or *dynamic system* frameworks (described below) to interrogate biological mechanisms may yield more scientific insight than continuing to rely on female-male categorical comparison.

Regardless of the number of categories employed, when operationalizing sex as a system of classification, there are several logical errors important to avoid. First, though categorical comparisons may enable the identification of certain group differences, the categories are not themselves causes or mechanisms, and so a categorical comparison itself can never *explain* what is driving any difference [11]. Relatedly, regardless of what characteristic(s) are used as a basis for sex categorization, it does not follow *that* specific characteristic itself is the mechanism, or can explain all sex-related variation in research outcomes, because there is some degree of correlation between many of the sex-associated characteristics (for example, genital morphology, reproductive organs and tissues, endogenous hormone levels, body composition) [60]. Thus, any interpretation of data based on sex categories must account for this. And finally, if the characteristic(s) we base our categorization on is a biological substance, it doesn’t follow that the placement into *categories* of female or male is inherently “natural” or even “scientific” (which is particularly relevant in the present political context where “Science” is often both weaponized and vilified).

To be sure, defining categories and comparing them is a valuable type of research design for many types of questions. At the same time, there is reason to be concerned about the heavy reliance on male-female categorical comparisons in response to institutional funding mandates to incorporate sex and gender considerations, particularly because of the likelihood that cultural stereotypes about women and men and rigid societal gender-based norms will influence the interpretation of data, including an over-emphasis on male-female difference [11, 25]. One potential benefit of the ongoing societal level debates about the nature of sex is that it can push us to ask ourselves what we think application of a certain categorical system of sex, in this case a male-female binary, is doing for us, and if there might be alternative ways to examine a phenomenon and develop more mechanistic insight than a male-female comparison allows.

### Sex as a trait

In contrast, operationalizing sex as a trait is enacted when a specific attribute is identified as being of relevance or interest. That specific attribute then serves as the factor used to frame investigation about the influence of *sex* in a given context. The history of science makes clear that which specific traits have been considered as defining or associated with sex have changed over time, often in relation to sociocultural changes and advances in science and technology [55, 61, 62]. Among the traits most commonly used are gamete size, chromosomal complement, gonad type, or genital morphology. But these could conceivably be any trait that is plausibly relevant to the project at hand. It is important to notice that a trait-based framework of sex does *not* itself require categorization into discrete groups, only the identification and measurement of the relevant trait. In other words, when applying a trait-based framework, the goal is not to choose a trait that we will use to define membership in a category, but rather to use the trait itself as the locus of analysis. For example, if serum testosterone level is identified as the trait of interest for investigation, the level is measured for each individual and the relationship between testosterone level and the outcome of interest is analyzed.

Again, gamete size is an emblematic example of a trait often used to operationalize sex; although typically understood as binary (i.e. large or small, ovum or sperm), both types of gametes can arise from the same individual at the same time (as in most types of plants and in some intersex individuals) or sequentially (in some vertebrate animals that can change the type of gametes they produce at different points in their life cycles), and so a binary view of gametes themselves does not necessarily require a binary view of organisms [54, 63]. Indeed, although some traits we associate with sex are binary in nature (e.g. the presence or absence of the SRY gene), others may come in some other number of states (e.g. the configuration of sex chromosomes: XX, XY, XO, XXY, XYY, XXX, etc.), some are more of a continuous distribution or spectrum (e.g. hormone levels), while others are more polymorphic (e.g. genital morphologies) or mosaic-like (e.g. brain structures) [64]. Rather than being considered only exceptions to a (binary) rule, these examples expose how varied ways of operationalizing sex, whether as bimodal clustering or a spectrum, are not in fact in conflict with one another. Instead, these are simply different ways of conceptualizing and making sense of the complexity of sex. In fact, reflection on these varied approaches within a single project, and even comparative analyses assessing their utility could expand our shared thinking about sex. In our view, a trait-based framework of sex is likely to be most useful when the trait itself is already known to be the mechanism of interest or highly proximal to it. For example, the use of gamete type to operationalize sex could be quite valuable in research or policy questions about some forms of contraception, but probably less useful in trying to understand the influence of sex on nociceptive pathways of pain. And, as noted with the issue of using sex as a system of classification noted above, because there is some degree of correlation between many sex-associated traits, researchers must be careful not to attribute causality unless the research design warrants it. We also must avoid the misapprehension that such a trait constitutes what sex ‘really is,’ particularly when it is operationalized uncritically.

### Sex as dynamic system

When this framework is guiding a given perspective or project, sex is understood as a collection of multiple elements that interact with one another, respond to stimuli, and change across the lifespan to produce a complex phenomenon that we regard as *sex.* Within this framework, sex is not defined by any single trait, but rather as the integration of a constellation of components acting on one another to produce emergent phenotypes, physiologies, and behaviours that we recognize as collectively constituting sex [65–67].

Using gamete size again as our starting point, in the dynamic systems framework of sex, gametes do not define sex but are recognized as one of many players in dynamic interaction to produce the complex phenomenon we call ‘sex’. The production of gametes themselves is understood to be regulated through the complex interplay of a variety of tissues, molecules, genes, and mediators, across multiple points in the lifespan. The organs and tissues that produce gametes develop through intricate developmental processes involving many genes and mediators; for example, although the SRY gene is often specified as the genetic origin of male-pattern development, recently published research has shown that SRY + mice can develop ovaries in the context of iron deficiency [68]. The physiological processes involved in gametic generation are governed by a network of hormones manufactured by a variety of organs throughout the body; the synthesis and release of those hormones is influenced by the various body mechanisms that maintain homeostasis and also by the individual’s exposure to external stimuli (physical, emotional, psychological, and otherwise) [69–72]. Secondary sex characteristics develop under the influence of both reproduction-related factors as well as other genes unrelated to reproduction per se along with external influences like nutrition, physical activity, and cultural influences. Reproduction-related processes have impacts on a wide range of body systems and functions (some of which induce temporary adaptations, while others are more persistent).

Unlike the trait or classification frameworks, applying a dynamic systems framework for conceptualizing sex tends to direct our attention not to individual elements themselves or categories to define it, but to the mechanisms *of interplay* between elements that produce variation in outcomes that we understand to constitute the complex phenomenon sex. Instead of trying to locate sex within any specific trait or categorizing individuals based on the presence of one or more these, a dynamic systems view sees sex as the emergent outcome of complex dynamic interaction between many traits and other factors.

It seems to us that this framework has considerable alignment both with entanglement views of sex/gender (as discussed previously) and also with the perspective that sex is not dimorphic. Like the entanglement of sex/gender, when sex is considered as a complex dynamic system it becomes impossible to pin down any one element as the locus of sex, neatly distinguish one category from another, or isolate sex from its context. This approach thus enables recognition that particular configurations of sex can vary considerably between two individuals, and even within the same individual at different times in the lifespan or in different contexts. This framework thus tends to be best for promoting thinking in terms of range of possibilities, acknowledging and embracing heterogeneity and diversity.

Understanding sex as a complex dynamic system is probably of particular value in research contexts where the goal is to understand the mechanisms through which sex- and gender-related variation is produced, because the systems concept itself is concerned with processes and interactions. At the same time, even when a dynamic system framework is being used as the conceptual frame, it may not be feasible to enact it in experimental design per se in many research contexts; thus in some cases the methodological operationalization may take the form of the classification or trait framework, but the dynamic system framework is the conceptual orientation applied to interpretation.

## Ways forward

Given the political weaponization of sex and science, it is more crucial than ever for scientists and scholars to be able to advance constructive cross-disciplinary dialogues about sex and gender. Here we suggest that the entrenched debate now itself risks undermining scientific collaboration and progress. We propose to eschew the *ongoing argument* in its current form, that pits binary versus non-binary operationalization of sex against one another in a continued impasse, and instead move our focus toward clarifying the varied aims, methods, frameworks and interpretations of data in scientific research, as others have also suggested (e.g. [6, 9, 73]). Toward that end, drawing on the frameworks we provide above, below we summarize a series of steps. We hope these enable nimble engagement with what sex is being leveraged *to do* and *why*, in order to then ask *how* a given conceptualization will advance learning about any given phenomena. We offer this series of questions and considerations to guide our critical read of scholarship and scientific engagement of sex in ways that can advance *cross-disciplinary dialogue and collaboration*. The aim here is to mitigate what sometimes fuels and underlies these debates (e.g., moralizing assumptions and (mis)interpretations) to instead bridge conceptual and disciplinary silos and help focus dialogue more constructively.

1. Draw on these three frameworks of sex (as a system of classification, as a trait, and as a dynamic system) to discern how a given scholar(s), scientist(s) or project is conceptualizing sex.
2. Reflect on what sex (however operationalized) is being used to *do.* What questions do the framework of sex employed enable to be addressed? How does this framing operate as part of a larger system?
3. Consider whether the framing and approach make sense for the research question and context.
4. When there is disagreement or critique - consider whether it is about *how* sex is operationalized (e.g. binary chromosomal sex, hormonal ratios, assigned sex at birth), or how sex is defined (e.g., gamete size, self-report), or if it is about something else (e.g., the aims or questions guiding the research).

An element of this that is key pertains to data interpretation and dissemination since this is the crucial way knowledge is created; *we recommend the dynamic systems framework is brought to bear during interpretive processes*: Even where the trait or system of classification framework of sex is used methodologically, we contend that a dynamic systems framework of sex should largely govern the way we think about the meanings, applications, and implications of our work, because the emphasis on mechanisms and networks and complexity makes it somewhat less susceptible to limiting sociocultural norms and stereotypes.

Methods of operationalization themselves should not stand in the place of specifying a rationale for a “way” of thinking about sex. Ideally, addressing the questions above as a series of steps, whether in one’s own research or when engaging that of others, can minimize refutation based on operationalization alone. But more importantly, scientists and scholars might gain insights into our own as well as one another’s research intentions. Toward bridging silos and outstanding debates, these steps could also clarify areas of consensus and dissensus. For example, epistemological differences about what kinds of questions *should* be asked pertaining to “sex differences” are clearly fueling a lot of the current debate; on the most extreme end, when there seems to be underlying assumptions grounded in biologically essentializing or reductionist notions of human behavior or capacity, the stand-off becomes political in nature. Though operationalization of sex is clearly a by-product of research goals and questions (and so in a way these recommendations become circular), we hope these frameworks and attendant suggestions contribute to a reorientation to help center the research aims, frameworks, and interpretations of the data instead of only specifics of operationalization. Reframing certain debates about the nature of sex toward dialogues that clarify research aims and attendant frameworks could further support rigor without rigidity in scientific research.

## Conclusion

We maintain that there is no universally applicable claim to whether sex is operationalized as binary, continuous, or is itself useful or useless as a concept. It is not the operationalization of sex in science itself that is the sole issue but how it is misused both scientifically *and* politically. We are not alone in these perspectives, but through these guiding frameworks and steps, we hope to provide a bridge between those “for” and those “against” binary thinking of sex in order to foster a diversity of approaches to the inclusion of sex and gender that build on one another rather than exist in opposition.

## Data Availability

No datasets were generated or analysed during the current study.

## Footnotes

1 A judicial ruling in June 2025 found that such cuts had in fact been made on a discriminatory basis and ordered the US federal government to restore funding; this restoration has not been enacted at the present time [21].
